# Cubes and playdough are feasible and acceptable methods to estimate food group intake with the Global Diet Quality Score app

**DOI:** 10.3389/fnut.2026.1908456

**Published:** 2026-09-03

**Authors:** Marieke Vossenaar, Mourad Moursi, Luka Pauwelyn, Joanne E. Arsenault, Winnie Bell, Megan Deitchler

**Affiliations:** 1 Intake—Center for Dietary Assessment, FHI 360, Washington, DC, United States; 2 FHI 360, Washington, DC, United States

**Keywords:** 3D cubes, diet quality, food group consumption, Global Diet Quality Score (GDQS) app, playdough

## Abstract

**Background:**

The Global Diet Quality Score application (GDQS app) enables standardized collection of dietary data across settings. The GDQS app allows for the use of 3-dimensional (3D)-printed cubes and playdough to estimate the amounts of food groups consumed for a 24-h reference period. This paper reports on the feasibility and acceptability of using a set of ten 3D cubes and playdough to estimate amounts consumed at the food group level among study participants.

**Methods:**

Enumerators conducted face-to-face interviews using the GDQS app with 170 study participants (71% female; aged 18–73 years) living in the Washington DC metropolitan area. The participants reported their consumption for the prior 24-h and used 3D cubes and playdough to estimate the amount of each food group consumed. The order in which participants were offered the cubes and playdough was randomized. Respondent feedback on use of the 3D cubes and playdough was collected through a 5-point Likert scale and open-ended questions.

**Results:**

Fewer than 2% of the study participants described either the cube or playdough method as ‘very difficult’. There was no statistically significant difference in preference between the two methods (45% preferred cubes and 55% preferred playdough, *p* = 0.167). The preference for method varied by food group. Some patterns emerged, such as a preference for cubes for estimating liquids consumed and playdough for estimating fruits and irregularly shaped foods.

**Conclusion:**

Both 3D cubes and playdough are feasible and acceptable methods for estimating GDQS food group intake, strengthening the evidence for the practicality of using the GDQS app for dietary data collection. Given the high acceptability of both methods, the choice of method—or the use of both in combination—for data collection should be guided by the operational context and resource availability. These findings reinforce the value of simplified, standardized tools for assessing diet quality across diverse field settings.

## 1 Introduction

The collection of data on diet quality enables policymakers to identify specific dietary improvements needed within populations to inform interventions and allocate resources. Ultimately, robust dietary data support the development of effective public health policies and interventions, fostering a healthier and more productive society.

The Global Diet Quality Score (GDQS) was developed to address the need for a simple, robust, and easily operationalized diet quality metric for global use and has been validated globally against nutrient intake and diet-related health outcomes among individuals 2 years and older (1–17). The GDQS is entirely food-based and does not require a food composition database or laborious processing of dietary data. The metric comprises 25 food groups: 16 classified as healthy, 7 as unhealthy, and 2 (‘red meat’ and ‘high-fat dairy’) considered unhealthy when consumed in excess. The tabulation of the GDQS requires estimation of the amount of each food group consumed over a 24-h reference period.

Portion size estimation is a well-recognized challenge in dietary surveys (18–20). In traditional quantitative 24-h dietary recalls, various types of portion size estimation methods are used to determine the grams of foods, beverages, and mixed dishes consumed. The GDQS does not require gram-based estimates of foods and ingredients as the metric scoring system awards points for consumption of each food group based on consumption ranges that have been defined for each food group (21). The consumption ranges are used to classify consumption as low, medium, or high (or very high for ‘high-fat dairy’) for each of the 25 food groups. Analyses conducted during the development of the GDQS metric revealed that incorporating quantitative intake data in the metric construction improved sensitivity to diet–outcome associations, with two cutoff values to define a consumption range (i.e., three categories of intake) outperforming a single cutoff value to define a consumption range (i.e., two categories of intake) (8). This improvement, however, relies on the availability of reliable and practical methods for estimating consumption.

To enable the standardized and efficient collection of GDQS data across settings, the *Intake—Center* for Dietary Assessment at FHI 360 developed the GDQS app (22). The GDQS app uses an open 24-h dietary recall method to record all foods, beverages, and ingredients consumed by the respondent during the previous day and night and relies on an integrated food database of more than 6,000 items for automated classification of each item consumed into the corresponding GDQS food group.

To eliminate the need for the respondent to estimate the gram amounts of individual foods, beverages, and ingredients consumed, ten 3-dimensional (3D) plastic cubes of predetermined size are used, each representing a different gram amount, on the basis of the volume of the 3D cube and the average estimated density of a given GDQS food group (23). Together, the ten 3D cubes represent the minimum and maximum gram intake values used by the GDQS metric scoring system for defining the low, medium, or high (or very high) consumption ranges for each food group. Evidence from Thailand showed that GDQS data collected with the GDQS app, with the set of ten 3D cubes used to estimate food group intake, yielded more associations with nutrient adequacy and metabolic risk indicators than GDQS data collected using conventional quantitative 24-h dietary recall interviews or food frequency questionnaires (24).

While 3D cubes offer a standardized approach for the estimation of food group intake, alternative methods could increase the accessibility of the GDQS app in settings where printing or procuring 3D cubes is challenging. Providing a second reliable option for estimating food group consumption amounts with the GDQS app would also have the benefit of offering increased flexibility for data collection methods, according to survey preference, and as appropriate for various cultural contexts and fieldwork conditions.

Playdough has emerged as a promising alternative for portion size estimation in quantitative 24-h dietary recall surveys, offering a simple, tactile, and interactive method that has been widely used in national dietary surveys in low- and middle-income countries (LMICs) (25–31). Its malleability makes it particularly useful for foods with irregular shapes or amorphous textures (18). Although traditionally applied to estimate individual food portions, given its wide-scale use, acceptability, and validity as a portion size estimation method in dietary surveys, its potential for estimating total amounts consumed at the food group level warranted exploration within the framework of data collection with the GDQS app.

Moursi et al. (23) evaluated the use of both 3D cubes and playdough for estimating food group amounts with the GDQS app, using weighed food records as the reference method. The results indicated that the GDQS scores derived from using either 3D cubes or playdough were equivalent to those obtained from weighed food records. The agreement for most of the GDQS food groups was substantial (Kappa 0.61–0.80) or almost perfect (above 0.81), with significant correlations observed for 22 and 23 out of 25 food groups for 3D cubes and playdough, respectively (*p* < 0.05).

Having demonstrated that both 3D cubes and playdough provide reliable estimates of the amounts of GDQS food groups consumed, this paper draws on additional data from the Moursi et al. (23) study to report findings on respondents’ perceptions of the feasibility and acceptability of each method.

## 2 Methods

### 2.1 Study design

As the study design has been described in detail elsewhere (23), only a brief overview is provided here. Data were collected from 170 adults living in or near Washington, DC, between November 2022 and June 2023 using a repeated-measures design. On day one of data collection, the participant recorded food consumption over 24-h at home using weighed food record paper forms developed for the study. The following day, the participant reported food consumption for the same 24-h period through an enumerator-administered interview using the GDQS app. During the GDQS app interview, 3D-printed cubes and playdough were provided in a randomized order to the participant for estimating the amount of each food group consumed. Immediately after completion of the GDQS app interview, the enumerator administered a semi-structured questionnaire to collect the participant’s feedback on the use of the 3D cubes and playdough. This paper focuses solely on the participant feedback data collected.

### 2.2 Data collection using the GDQS app

The GDQS app runs on Android devices and must be administered by a trained enumerator (it cannot be self-administered). The GDQS app functions fully offline. The GDQS app uses an open-recall approach, in which the enumerator asks participants to report all foods, beverages, and mixed dishes consumed during the previous 24-h. For any mixed dish mentioned, the GDQS app prompts the enumerator to collect ingredient information. Additional prompts provided by the GDQS app help categorize foods, beverages, and ingredients into their appropriate GDQS food groups—for example, probes to distinguish between low-fat and high-fat cow milk to make the corresponding classification in the low- or high-fat dairy GDQS food group. The process also includes recording items such as purchased deep-fried foods, liquid oils added to dishes, and caloric sweeteners. In the final step of data collection, the GDQS app automatically sorts all foods, beverages, and ingredients reported as consumed by GDQS food group and prompts the enumerator to ask the participant to estimate the total amount consumed of each food group.

The GDQS app normally uses a set of ten 3D cubes to estimate food group intake. These 3D cubes must be physically available during data collection, as two-dimensional images or photographs are insufficient for accurately visualizing volume. For this study, the GDQS app was adapted to also allow the use of playdough to estimate food group intake. The GDQS app was programmed to randomize the order of the method used to estimate food group intake, with participants first estimating all food groups using either 3D cubes or playdough and then repeating the estimations using the alternative method.

#### 2.2.1 Using cubes to estimate food group intake

The 3D cubes were placed in front of the study participant in order of size, from cube number 1 (smallest) to cube number 10 (largest). Using automated prompts from the GDQS app, the enumerator reminded the participant of the foods, beverages, and ingredients consumed for each GDQS food group. After reading the list of foods, beverages and ingredients consumed for a given food group, the enumerator then asked the participant to visualize the combined amount of foods, beverages, and ingredients consumed within that food group and to point to the cube closest in size (volume) to the total amount consumed. The GDQS app allows for the selection of only one of the 10 cubes for each GDQS food group. It is not possible to select fractions or multiples of any of the ten cube sizes. If the amount consumed for a food group was larger than the volume of cube 10 (the largest cube), the participant was instructed to select cube 10. This procedure was repeated for all the GDQS food groups reported as consumed by the respondent, except for liquid oils. The amount of liquid oils consumed is not estimated by the respondent; instead, the GDQS app uses an algorithm to infer the range of consumption (23).

The GDQS app uses the cube size selected by the respondent to classify food group consumption into the appropriate consumption ranges of intake (i.e., low, medium, high, or very high). To do this, the GDQS app calculates the respondent’s gram intake for the food group from the known volume of the cube selected and the estimated average density of the food group and then automatically assigns a consumption range for that food group.

#### 2.2.2 Using playdough to estimate food group intake

The participants were given a large amount of playdough (approximately 4 kg). Using automated prompts from the GDQS app, the enumerator reminded the participant of the foods, beverages, and ingredients consumed for each GDQS food group. After reading the list of foods, beverages and ingredients consumed for a given food group, the enumerator then asked the participant to visualize the combined amount of foods, beverages, and ingredients consumed within that food group and to mold the playdough into the general shape and size that represents the total amount consumed. The enumerator then weighed the playdough the respondent had molded using a calibrated electronic dietary scale (My Weigh® KD7000) and recorded the weight to the nearest gram in the GDQS app. The participants were allowed to mold every single food, beverage, or ingredient separately and then to combine the amounts of playdough or to mold the total amount of playdough to correspond to the volume of all foods, beverages, and ingredients consumed in the food group. This procedure was repeated for all GDQS food groups consumed, except for liquid oils.

When using playdough, consumption ranges of intake (i.e., low, medium, high, or very high) for each food group were estimated based on the amount of playdough molded by the respondent and weighed by the interviewer. To do this, the GDQS app calculates the respondent’s gram intake for the food group from the weight of the playdough and the estimated average density of the food group and then automatically assigns a consumption range for that food group.

#### 2.2.3 Time needed to complete data collection with the GDQS app

For the study, the GDQS app was adapted to create time stamps at the start and end of each interview and at the start and end of the step used to estimate food group intake using each method. This allowed us to derive the time of the entire interview using either playdough or 3D cubes, and the time for each method to estimate food group intake. The time stamps were created partway through the study and in some cases interview time was not stopped at the end of the interview. As a result, reliable time data is available only for 103 of the 170 interviews completed.

### 2.3 Participant feedback on the use of cubes and playdough

After data collection with the GDQS app was completed, the enumerators administered a semi structured questionnaire to collect participant feedback on the use of the 3D cubes and playdough to estimate the amount of each food group consumed.

A 5-point Likert scale was used to rate how difficult or easy each method was to estimate food group intake (1 = very difficult, 5 = very easy). The 3D cubes and playdough were rated separately, following the same order in which the methods were presented for use by the participant. For both methods, all participants were then asked open-ended questions about the most difficult and easiest aspects of each method. The question about the most difficult aspect was asked first, followed by the question about the easiest aspect.

Next, the participants were asked whether there were any specific foods or beverages or groups of foods or beverages for which estimating the consumed amounts was particularly difficult or easy, and those who answered affirmatively were prompted to describe the relevant items. Finally, all the participants were asked which method they preferred for estimating food group intake (3D cubes or playdough) and to explain the reasons for their preference.

### 2.4 Data analysis

Descriptive statistics were used to tabulate the Likert scale responses and open-ended feedback. A chi-square test was used to assess whether there was a statistically significant difference in the preference for method (3D cubes or playdough) across study participants. The open-ended qualitative responses were categorized into common themes or topics by two independent researchers (MV and LP). Where there were discrepancies, MV made the final decisions. For each question, all the responses were examined to develop a consistent coding system using labels such as “large gap between cubes,” “similarly sized cubes,” and “difficulty visualizing shape”. The foods and beverages reported were grouped into similar types of foods, such as “nuts and seeds”, “beans and peas”, “noodles, spaghetti, and pasta”, “sauces, dips, spreads”, “vegetables”, “fruits”, and “liquids and beverages”. Once the labels were created, the responses within each theme or topic were recorded. All respondent feedback analyses were conducted using Microsoft Excel.

Descriptive statistics for average total GDQS app interview time using either 3D cubes or playdough and time needed to estimate amounts consumed by each method were tabulated. Mean differences in interview times were examined using Paired *T*-tests. In addition, Spearman’s correlations were used to examine the relationship between time spent using a method and its ease-of-use rating (Likert scale responses). All statistical tests were conducted using SAS (Version 9.4).

### 2.5 Ethical considerations

FHI 360’s Office of International Research Ethics reviewed and approved the study. It was determined that the research qualifies for one or more exempt categories according to the U.S. federal regulations known as the Common Rule 45 CFR 46.104(d) due to its low-burden, minimal-risk procedures. All study participants provided written informed consent and received a $200 electronic gift card for participation in the full study.

## 3 Results

### 3.1 Demographic information of the study participants

Descriptive details about the study population are provided in Table 1. In brief, the study participants ranged in age from 18 to 73 years, were predominantly female (71%), had diverse ethnic and educational backgrounds, and lived mostly in households of two or more people (79%).

**Table 1:** Demographic information of the study participants (*n* = 170).

Characteristics	*n*	%
Age groups		
18–24 year	48	28.2
25–35 year	69	40.6
36–45 year	21	12.4
46–73 year	32	18.8
Sex		
Male	48	28.2
Female	121	71.2
Refused to answer	1	0.6
Ethnicity		
White	86	50.6
Asian	40	23.5
Black or African American	33	19.4
American Indian or Alaska Native	1	0.6
Other	10	5.9
Hispanic/Latino		
Yes	20	11.8
Number of people living in the household		
1	36	21.2
2	43	25.3
3	33	19.4
4 or more	58	34.1
Highest level of education completed		
Some college (not completed)	26	15.3
Completed college	55	32.4
Some postcollege coursework	36	21.1
Completed college and a postcollege degree	53	31.1

### 3.2 Difficulty and ease of using cubes and playdough

#### 3.2.1 Most difficult and easiest aspects of using cubes and playdough

The participants’ feedback on the most difficult and easiest aspects of using 3D cubes and playdough is summarized in Table 2.

**Table 2:** Participants’ feedback on the use of playdough and cubes to estimate amounts of food groups consumed using the GDQS app^a^.

	Feeback	*n*	% of study participants (*n* = 170)
Cubes			
Most difficult part	Visualizing the shape and/or volume of the foods as a cube	87	51.2
	The large difference in size between cubes 8 and 9	68	40.0
	When the amount of foods consumed was between two cube sizes	15	8.8
	Visualizing the volume of several foods combined within a food group	14	8.2
	Some cubes were similarly sized, making the choice difficult	7	4.1
	Remembering how much was consumed	5	2.9
	Foods are served in household utensils, not cubes	5	2.9
Easiest part	Cubes helped visualize the amounts of foods and beverages consumed	89	52.4
	Equating the cubes to household measuring cups and bowls	23	13.5
	Having an array of sizes of cubes laid out	19	11.2
	Choosing a cube size for small amounts of foods	16	9.4
	Choosing a cube size for large amounts of foods	11	6.5
	Choosing from a predetermined set of cubes	11	6.5
	Visualizing putting foods and beverages into the cubes	9	5.3
	When the volume of food consumed was similar to a cube size	4	2.4
Playdough			
Most difficult part	The difference in weight between playdough and foods	68	40.0
	Molding the playdough to the appropriate shape	46	27.1
	Representing amounts of liquids consumed	20	11.8
	Remembering how much was consumed	19	11.2
	Visualizing the volume of foods consumed	17	10.0
	Visualizing the volume of several foods combined within a food group	17	10.0
	Difference in shape and texture between foods and playdough	14	8.2
	Estimating small amounts of foods	8	4.7
	Estimating large amounts of foods	6	3.5
Easiest part	Molding playdough into different shapes and sizes	105	61.8
	Playdough provides a visual representation of foods consumed	31	17.6
	A hands-on and tactile approach	22	12.9
	Allows the amount to be adjusted	8	4.7
	Fun to use	4	2.4
	Allows molding foods separately	3	1.8
	Allows molding of small foods	3	1.8

^a^Questions were open ended and study participants were allowed to provide up to three responses.

Approximately half of the participants found it difficult to match the shape or volume of food to a cube, and many reported that the gaps between available cube sizes were either too large or too small, making selection challenging. Additional difficulties reported by participants included choosing a cube when the consumed amount fell between two sizes, visualizing the volume of several foods combined, recalling how much was eaten, and the mismatch between cube representations and typical household measures.

In contrast, approximately half of the participants reported that the cubes helped them visualize the amounts consumed, and some found it straightforward to link cube volumes to familiar household measures such as bowls or measuring cups. Several participants also appreciated having a predefined set of cubes laid out in front of them, and some noted that cubes worked well for both very small and very large portions.

Nearly half of the participants reported difficulty using playdough because of the difference in weight between playdough and the foods consumed. Some participants also struggled with the differences in shape and texture between playdough and the foods consumed. About one-third of the participants found it hard to mold playdough into the actual shape of the food and to account for spaces between items (e.g., potato chips). Other challenges reported by participants included representing liquids with a solid material, visualizing foods consumed across different meals, remembering how much was eaten, estimating the volume of foods, and, for a few participants, representing very small or very large amounts with playdough.

Almost two-thirds of participants found it easy to mold playdough into different shapes and sizes, and many described playdough as a helpful method to provide a visual representation of the amount consumed. Several participants enjoyed the hands-on, tactile nature of this method, and a few highlighted the specific advantages of using the playdough method, such as adjusting the amount by adding or removing playdough, molding different foods separately, and representing small items.

#### 3.2.2 Foods that were especially difficult or easy to estimate

The ease of estimation of the quantity consumed varied by food or beverage type. Although more than two-thirds of the participants found certain items challenging to estimate using either cubes (70.0%) or playdough (81.2%), the majority reported that there were items that were especially easy to estimate with either cubes (88.8%) or playdough (89.5%).

Table 3 lists types of foods that were particularly difficult or easy to estimate with 3D cubes and playdough.

**Table 3:** Foods and beverages that were particularly difficult or easy to estimate using cubes or playdough^a^.

	Food items	*n*	% of total participants (*n* = 170)^b^	% of participants asked to report type of food^c^
Cubes				
Number of respondents who reported that certain food items were especially difficult to estimate		119	–	–
Types of foods that were difficult	Vegetables	37	21.8	31.1
	Bread, bagels, toast, tortillas	27	15.9	22.7
	Poultry, fish, red meat	16	9.4	13.4
	Beverages	15	8.8	12.6
	Fruits	14	8.2	11.8
	Cheese, cream, yogurts	13	7.6	10.9
	Mixed dishes	10	5.9	8.4
Number of respondents who reported that certain food items were especially easy to estimate		151	–	–
Types of foods that were easy	Beverages	34	20.0	22.5
	Fruits	25	14.7	16.6
	Vegetables	24	14.1	15.9
	Cheese, cream, yogurts	20	11.8	13.2
	Cakes, cookies, sweets	20	11.8	13.2
	Poultry, fish, red meat	19	11.2	12.6
	Bread, bagels, tortillas	18	10.6	11.9
	Noodles, spaghetti, pasta	17	10.0	11.3
	Sauces, dips, spreads	17	10.0	11.3
	Rice	16	9.4	10.6
	Nuts, seeds	10	5.9	6.6
	Sugar, honey	10	5.9	6.6
	Granola, cereals	9	5.3	6.0
Playdough				
Number of respondents who reported that certain food items were especially difficult to estimate		138	–	
Types of foods that were difficult	Vegetables	54	31.8	39.1
	Beverages	39	22.9	28.3
	Cheese, cream, yogurts	18	10.6	13.0
	Noodles, spaghetti, pasta	14	8.2	10.1
	Granola, cereals	13	7.6	9.4
	Chips, popcorn, pretzels	10	5.9	7.2
	Poultry, fish, red meat	9	5.3	6.5
	Sauces, dips, spreads	9	5.3	6.5
Number of respondents who reported that certain food items were especially easy to estimate		153	–	
Types of foods that were easy	Bread, bagels, toast, tortillas	57	33.5	37.3
	Fruits	52	30.6	34.0
	Poultry, fish, red meat	43	25.3	28.1
	Vegetables	33	19.4	21.6
	Cakes, cookies, sweets	27	15.9	17.6
	Cheese, cream, yogurts	19	11.2	12.4
	Eggs	17	10.0	11.1
	Noodles, spaghetti, pasta	10	5.9	6.5
	Beverages	10	5.9	6.5
	Granola, cereals	9	5.3	5.9

^a^Questions were open-ended, and participants could provide up to three responses.

^b^Only foods reported by at least 5% of participants are shown.

^c^Only participants who indicated that certain food items were especially difficult or easy to estimate were asked to respond.

The participants reported difficulty in estimating the amounts of oddly shaped items with cubes, particularly leafy vegetables (e.g., lettuce, spinach), irregular vegetables (e.g., avocado, tomato, broccoli), breads and other grain products (e.g., bread, bagels, tortillas), and meats (e.g., poultry, fish, red meat). The perceptions of fruits and vegetables varied both by type and by participant—some items were often described as difficult to estimate with cubes (e.g., leafy greens, tomatoes), whereas others were generally considered easier (e.g., apples, berries, oranges, lettuce, broccoli, and avocado). Beverages were generally considered easy to visualize using cubes.

When using playdough, participants reported difficulties in representing vegetables—particularly leafy greens (e.g., lettuce and spinach) and irregularly shaped items such as broccoli. The perceptions of the ease of using playdough to estimate vegetables, however, varied by both type and participant, as some respondents found them relatively easy to estimate (e.g., avocado, tomato). Beverages were consistently described as difficult to represent by molding a solid material. In contrast, breads and tortillas, fruits (e.g., bananas, apples, berries), and meats were generally considered easy to visually represent with playdough.

#### 3.2.3 Reasons why some foods are especially difficult or easy to estimate

Table 4 lists the reasons why some types of foods were particularly difficult or easy to estimate with cubes and playdough.

**Table 4:** Reasons why some foods and beverages were especially difficult or easy to estimate using cubes or playdough^a^.

	Reasons	*n*	% of total participants (*n* = 170)^b^	% of participants asked to report reason^c^
Cubes				
Number of respondents who reported that certain food items were especially difficult to estimate		119	–	–
Reasons why it was difficult	Difficult to visualize the shape and/or volume of the foods as a cube	76	44.7	63.9
	The large difference in size between cubes 8 and 9	22	12.9	18.5
	Foods have air or spaces in between	14	8.2	11.8
	Visualizing the volume of several foods combined within a food group	13	7.6	10.9
Number of respondents who reported that certain food items were especially easy to estimate		151		
Reasons why it was easy	Cubes helped visualize the amounts of foods consumed	87	51.2	57.6
	Cubes are like household bowls, measuring cups, and containers	30	17.6	19.9
	Small cube sizes allow estimating small amounts of foods	24	14.1	15.9
	Large cube sizes allow estimating large amounts of foods	20	11.8	13.2
	Shape of the food matched the shape of the cube	17	10.0	11.3
Playdough				
Reported that certain food items were especially difficult to estimate		138		
Reasons why it was difficult	Difficult to visualize and/or mold playdough into the shape of the food	50	29.4	36.2
	Difficult to estimate liquids using solid playdough	33	19.4	23.9
	Difference in weight/density between playdough and foods	24	14.1	17.4
	Foods have air or spaces in between	15	8.8	10.9
	Difficult to mold several portions of small foods	15	8.8	10.9
	Food has no defined shape	13	7.6	9.4
	Difficult to mold volume of several foods combined within a food group	11	6.5	8.0
	Difficult to remember amounts consumed	8	4.7	5.8
	Large volume	7	4.1	5.1
Reported that certain food items were especially easy to estimate		153		
Reasons why it was easy	Easy to mold into the shape of the food	108	63.5	70.6
	Easy to visualize	22	12.9	14.4
	Food had a standard shape	18	10.6	11.8
	Remember amount consumed	11	6.5	7.2
	The food was a single item	10	5.9	6.5
	The food was small	10	5.9	6.5

^a^Questions were open-ended, and participants could provide up to three responses.

^b^Only foods reported by at least 5% of participants are shown.

^c^Only participants who indicated that certain food items were especially difficult or easy to estimate were asked to respond.

The participants who experienced difficulty in estimating particular foods cited challenges such as visualizing irregularly shaped items as 3D cubes (e.g., vegetables, bread, chicken, fruits, cheese), the large gap between available cube sizes (e.g., cubes 8 and 9), accounting for gaps among foods such as potato chips, cereals, and lettuce, and determining total volumes when combining multiple foods across different meals or recipes. In contrast, participants who found certain foods straightforward to estimate using cubes frequently indicated that the cubes facilitated the visualization of quantities, bore resemblance to common measuring instruments, or were suitable for assessing both small and large portions—particularly for foods with shapes analogous to cubes.

Reported challenges using playdough included difficulties in shaping playdough to accurately represent food forms, estimating liquid quantities with a solid medium, addressing density differences for lighter foods such as lettuce, spinach, tortilla chips, and popcorn, representing foods with spatial gaps (e.g., lettuce, broccoli), managing numerous small food items (e.g., tortilla chips, beans), amorphous items (e.g., parmesan cheese, liquids), combinations of foods within groups, and estimating larger quantities. For participants who reported ease in estimating foods with playdough, many emphasized the advantage of being able to mold playdough into the shapes of various foods, whereas others noted that it was particularly easy to mold foods that have a standard shape.

### 3.3 Overall acceptability of cubes and playdough

**Figure 1:**
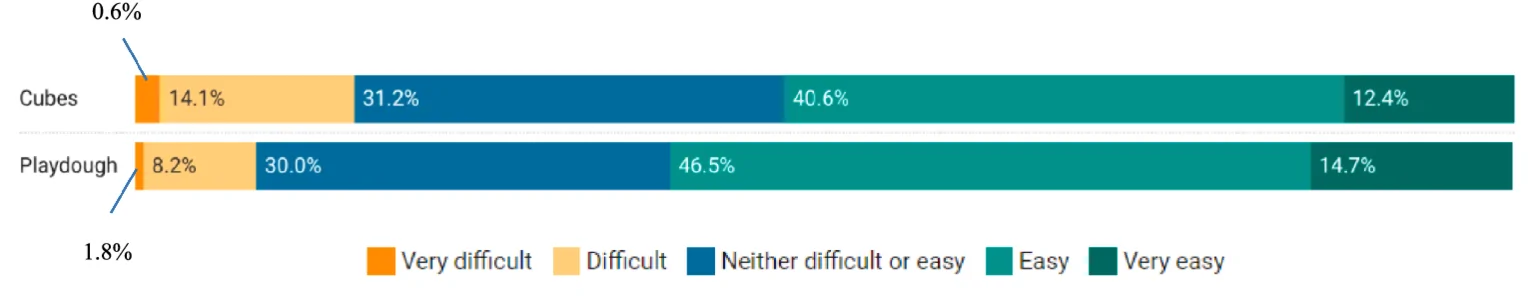
Ease of using cubes and playdough to estimate amounts (*n* = 170). The participants were asked to describe how easy or difficult it was to indicate (i) which cube was the closest in size to the amount of different foods and beverages consumed and (ii) the amount of different foods and beverages consumed using the playdough, using a scale of 1 (very difficult) to 5 (very easy).

Regarding overall acceptability, more than half of participants rated the 3D cubes (53%) and playdough (61%) ‘easy’ or ‘very easy’ to use (Figure 1), whereas approximately a third rated both methods as ‘neither difficult nor easy’. Few found playdough (9%) or cubes (16%) ‘difficult’ or ‘very difficult’ to use, and fewer than 2% considered either method ‘very difficult’. These findings suggest a generally high level of acceptability of both methods in terms of ease of use.

### 3.4 Preference for method to estimate amounts

Among the participants, there was no statistically significant difference in preference for the method used to estimate food group intake: 45% preferred using cubes, whereas 55% favored playdough (*p* = 0.167). The participants’ reasons for preferring either playdough or cubes are listed in Table 5.

**Table 5:** Participants’ reasons for their preference for playdough or cubes to estimate amounts of food groups consumed using the GDQS app.

	Reasons	*n*	% of participants who preferred method^a^
Respondent preferred cubes		77	–
Reasons why cubes were preferred	Easier to visualize the amounts consumed	33	43%
	Cubes are easier to use	22	29%
	Cubes provide set options with predetermined sizes	15	20%
	Cubes resembled cups, bowls, and other containers used in the house	14	18%
	Not having to create something from scratch or manually	9	12%
	The use of cubes was quicker	2	3%
	Less messy than playdough	2	3%
Respondent preferred playdough		93	–
Reasons why playdough was preferred	Could mold the playdough into the shape of foods	41	44%
	Easier to visualize the amounts consumed	33	36%
	Able to touch and handle playdough	19	20%
	Playdough was more accurate	15	16%
	Not bound to set sizes	13	14%
	Had more control and greater flexibility	8	9%
	Were able to add and remove bits of playdough	2	2%
	Was able to estimate foods separately	2	2%

^a^Only participants who preferred the method were asked to respond.

Among the participants who favored cubes, frequent reasons were that cubes made amounts consumed easier to visualize, were simpler to use, provided predetermined sizes, and resembled common household items such as cups, bowls, or containers.

Among participants who preferred playdough, common reasons included the ability to mold it into shapes of various foods, easier visualization of consumption amounts, and appreciation for the tactile aspect of handling the material, along with perceptions of increased accuracy, lack of restriction to set sizes, and greater control and flexibility.

### 3.5 Interview duration for data collection with the GDQS app using cubes versus playdough

The average interview time with the GDQS app was 15.9 (7.2) minutes using cubes, whereas it was 20.3 (8.3) minutes with playdough (Table 6). Estimating the amount of each food group consumed took participants an average of 4.2 min with cubes and 8.6 min with playdough, a statistically significant difference (*p* < 0.001). There was no association between time spent using playdough and its ease-of-use rating (Spearman’s 𝜌 = −0.03, *p* = 0.773), whereas a significant negative association was observed for time spent using cubes and its ease-of-use rating (Spearman’s 𝜌 = −0.26, *p* = 0.008).

**Table 6:** Interview duration for data collection with the GDQS app using cubes versus playdough to estimate the amounts consumed (*n* = 103)^a^.

Time	Using cubes to estimate amounts consumed		Using playdough to estimate amounts consumed		*p*-value for difference between cubes and playdough^b^
	Mean	SD	Mean	SD	
Total interview time (min)	15.9	7.2	20.3	8.3	<0.001
Time needed to estimate food group intake (min)	4.2	2.3	8.6	4.2	<0.001

^a^The interview time was not recorded for the first 42 interviews because the GDQS app was adapted to collect interview time after the study had already begun. In addition, 25 interviews were excluded because the interview time was not stopped at the end of the interview.

^b^Paired *T*-test for mean differences.

## 4 Discussion

The study participants reported that both 3D cubes and playdough were feasible and acceptable methods for estimating the amounts of food groups consumed, with fewer than 2% describing either method as “very difficult.” These findings are consistent with those of a feasibility study of the GDQS app using cubes conducted in Ethiopia among women who reported a positive experience using the cubes, and most (78%) found it easy to select the cube corresponding to the amount consumed from each food group (32). The positive participant feedback corroborates findings from the validation study led by Moursi et al. (23), which showed substantial to almost perfect agreement between estimates using 3D cubes or playdough and weighed food records for most GDQS food groups, indicating that these materials are not only valid for estimating food group quantities but also well accepted by study participants.

Among the study participants, there was no statistically significant difference in preference between the two methods used to estimate food group intake (45% preferred 3D cubes, and 55% preferred playdough). The preference between methods varied between food types, but no strong patterns emerged. Approximately one-fifth of the participants considered vegetables and liquids difficult to estimate with both methods. Nevertheless, the validation study led by Moursi et al. (23) showed substantial to almost perfect agreement between cubes and playdough against weighed food records for cruciferous vegetables, deep orange vegetables, other vegetables, sugar-sweetened beverages, and juices. For dark green leafy vegetables, the agreement was moderate for cubes and substantial for playdough.

Both cubes and playdough allow consumption to be categorized into low, medium, high, or very high ranges, which is sufficient for GDQS tabulation. Cubes provide a set of fixed shapes and sizes, whereas playdough offers greater flexibility because it can be shaped to match the volume of consumed food groups for more precise quantity estimates.

The use of 3D cubes and playdough to estimate the portion sizes of individual foods, beverages, and mixed dishes was found acceptable by study participants in various settings (19, 28, 31, 33). Playdough, in particular, has been widely used for portion size estimation in quantitative dietary surveys in LMICs, including recent large-scale and nationally representative quantitative 24-h dietary recall surveys in Niger (30), Nigeria (25) and Zambia (27). Although cubes are less commonly used for portion size estimation, Bucher et al. (33) developed a cube with a total volume of 64 cm^3^, which can be divided into eight smaller subcubes (each measuring 2 × 2 × 2 cm). The cube developed proved useful for estimating food volume and performed well compared with direct weight. However, participants found it challenging to estimate the portion sizes of foods that did not resemble the cube shape, especially irregularly shaped foods such as French fries.

The estimation of the amounts of food groups consumed—instead of individual food items—is an innovative concept. To our knowledge, the feasibility of using playdough to estimate the amounts of food groups has not been explored before. In this study, approximately 10% of the participants reported finding it difficult to visualize the volume of several foods combined within a food group, regardless of the method used.

The results of this study indicate that both 3D cubes and playdough are viable methods for estimating portion sizes, with no statistically significant difference in overall preference between methods. The method(s) selected for use with the GDQS app should therefore be determined by logistical factors relevant to the local setting. The GDQS app currently (September 2026) supports the use of cubes as the only method to estimate food group intake but will soon be available to also allow the use of playdough.

The duration of the interview is an important factor when choosing a method to estimate food group intake. In this study, using cubes to estimate food group amounts was more time-efficient (an average of 4 min per respondent for all food groups consumed) than using playdough (an average of nearly 9 min per respondent for all food groups consumed) (*p* < 0001). This difference was expected, because a respondent pointing to a cube that corresponds to a given amount is inherently faster than the task of a respondent molding playdough to the volume of food consumed and an enumerator weighing that volume of playdough. However, the actual time savings will depend on the study context and on the complexity of the diet being assessed.

Despite taking longer, playdough received higher ease-of-use ratings from respondents (61% rated playdough as “easy” or “very easy” versus 53% for cubes). Moreover, there was no association between the time spent using playdough and its ease-of-use rating (Spearman’s 𝜌 = −0.03, *p* = 0.773). These findings suggest that perceived ease reflects subjective aspects—such as a sense of control, flexibility in shaping portions, and the enjoyment or familiarity of handling playdough—rather than the speed of obtaining estimates. Ultimately, whether this time difference is meaningful in practice will depend on how much interview duration constrains the logistics of the study. In addition, cubes offer practical advantages in field settings, as they are lightweight, easy to store and transport, and can be reused in future studies.

In contrast, playdough is heavier, requires sealed storage containers and close monitoring of density during data collection, and has a limited shelf life even under proper storage conditions (34). Furthermore, digital dietary scales are required for weighing playdough, must be placed on stable surfaces for accurate readings and require routine checks for proper operation (35). Another important aspect is the cost and practicality of sourcing high-quality playdough and reliable digital dietary scales versus 3D-printed cubes. High-quality scales and playdough often need to be acquired from outside the country where data will be collected, which can complicate logistics due to potential import procedures. In comparison, plastic 3D cubes can typically be printed domestically at an estimated cost of 10–30 USD per set. To date, 3D cubes with specifications for the GDQS app have been printed and used successfully in Bangladesh, Benin, Cameroon, Colombia, Ethiopia (32), India, Jordan, Kenya, Lebanon, Mexico, Myanmar, Nepal, Nigeria, the Philippines, Singapore, Tanzania, Thailand (24), and the United States of America (23).

In settings where the logistical context allows, the GDQS app will be able to incorporate both 3D cubes and playdough estimation methods within the same survey. This flexibility will enable study participants to select the most suitable method for each GDQS food group—for example, using cubes for the GDQS food group, ‘juices’ and playdough for the GDQS food group, ‘other fruits’—accommodating individual visualization preferences and food-specific challenges while maintaining standardized data quality.

The strong performance and acceptability of both the 3D cubes and playdough for estimating amounts of food group consumed reinforce the value of using a purpose-built data collection tool and address a key concern of innovative portion size estimation methods among new users. A study in Thailand (24) showed that the GDQS app using 3D cubes robustly captured nutrient adequacy and noncommunicable disease–related outcomes, while offering several practical advantages over food frequency questionnaires (FFQs) and 24-h dietary recalls. GDQS app administration took approximately 10–20 min, compared with 30–35 min for 24-h dietary recalls and 45–60 min for FFQs. The GDQS app was also less cognitively demanding for participants, and lightweight, stackable cubes were more portable than the materials required for the GDQS other methods. Finally, whereas 24-h dietary recalls and FFQ data processing was relatively labor-intensive, GDQS app data processing was automatic.

A key strength of this study was its randomized within-person comparison of two GDQS app portion-size methods, combined with both semi-quantitative and qualitative participant feedback. Important limitations include the convenience sample from a U.S. metropolitan area, the demographic skew toward women, the relatively high level of schooling, and the limited applicability to routine field conditions because data collection differed from the settings in which the GDQS app would typically be used. As such, these findings may be limited in generalizability. Nevertheless, we believe that this formative validation study provides rich insights into the potential viability and ease of use of a simplified quantity estimation method. The core design features of the GDQS cubes (simple shapes, limited number of sizes, and direct visual comparison) were explicitly developed to be usable in low-literacy and low-education settings. The acceptability of the GDQS app and 3D cubes is further supported by the feasibility study conducted in Ethiopia among women from two diverse population groups, including many with limited formal education (32).

## 5 Conclusion

This study adds to the evidence base supporting the feasibility and acceptability of the GDQS app for data collection by demonstrating that both cubes and playdough are feasible and acceptable tools for estimating GDQS food group intakes. Strong validation evidence already exists to support their accuracy for food group intake estimation relative to weighed food records (23). Although playdough allows flexible volume shaping, cubes offer logistical advantages in cost, portability, and time efficiency, making them particularly suitable for large-scale surveys. Given these findings, the choice of method—or the use of both methods in combination—should be guided by the operational context and resource availability, as participant acceptability was high for both methods. Together with prior evidence from multiple settings, including the successful use of GDQS cubes with surveys carried out with the GDQS app in 18 countries across Africa, Asia, South America, and North America, these results reinforce the practicality of simplified, standardized tools for dietary data collection to assess diet quality and support their continued use and adaptation in diverse field contexts.

## Data Availability

The raw data supporting the conclusions of this article will be made available by the authors, without undue reservation.
